# Teleost Fish and Organoids: Alternative Windows Into the Development of Healthy and Diseased Brains

**DOI:** 10.3389/fnmol.2022.855786

**Published:** 2022-08-11

**Authors:** Giulia Fasano, Claudia Compagnucci, Bruno Dallapiccola, Marco Tartaglia, Antonella Lauri

**Affiliations:** Genetics and Rare Diseases Research Division, Ospedale Pediatrico Bambino Gesù, Istituto di Ricovero e Cura a Carattere Scientifico (IRCCS), Rome, Italy

**Keywords:** forebrain, cortical malformations, rare neurodevelopmental diseases, teleosts, organoids

## Abstract

The variety in the display of animals’ cognition, emotions, and behaviors, typical of humans, has its roots within the anterior-most part of the brain: the forebrain, giving rise to the neocortex in mammals. Our understanding of cellular and molecular events instructing the development of this domain and its multiple adaptations within the vertebrate lineage has progressed in the last decade. Expanding and detailing the available knowledge on regionalization, progenitors’ behavior and functional sophistication of the forebrain derivatives is also key to generating informative models to improve our characterization of heterogeneous and mechanistically unexplored cortical malformations. Classical and emerging mammalian models are irreplaceable to accurately elucidate mechanisms of stem cells expansion and impairments of cortex development. Nevertheless, alternative systems, allowing a considerable reduction of the burden associated with animal experimentation, are gaining popularity to dissect basic strategies of neural stem cells biology and morphogenesis in health and disease and to speed up preclinical drug testing. Teleost vertebrates such as zebrafish, showing conserved core programs of forebrain development, together with patients-derived *in vitro* 2D and 3D models, recapitulating more accurately human neurogenesis, are now accepted within translational workflows spanning from genetic analysis to functional investigation. Here, we review the current knowledge of common and divergent mechanisms shaping the forebrain in vertebrates, and causing cortical malformations in humans. We next address the utility, benefits and limitations of whole-brain/organism-based fish models or neuronal ensembles *in vitro* for translational research to unravel key genes and pathological mechanisms involved in neurodevelopmental diseases.

## Introduction

Since the early comparative studies in animal models (i.e., mouse, chick, and fish), developmental biologists have shed light on the precise choreography of genetically controlled events that shape the vertebrate brain in different domains along the AP (antero-posterior) and D-V (dorso-ventral) axes. These events produce the conserved organization of the forebrain anteriorly, morphologically and functionally distinguished in different domains (Puelles, 1995; Puelles et al., 2000). The pallium originates from the dorsal division of the anterior most part of the forebrain (telencephalon) and gives rise to the neocortex in mammals (Haines and Mihailoff, 2018); responsible for the high computation capacity beyond sensory-motor integration, such as language and abstract thinking skills.

Within the developing pallium, a precise balance and timing of signaling drive neural fate commitment and patterning, as well as the rate of progenitor pools’ expansion (level 1), migratory behavior and differentiation (level 2), and the establishment of connectivity patterns (level 3). Fine changes in local patterning schemes and precursor cells’ behavior across evolution underlie the array of diversity in vertebrate cognitive capabilities and the formation and expansion of the multilayered cytoarchitecture of the mammalian cortex, responsible for high-order functions typical of humans (Striedter, 2005; Van Essen et al., 2018; Stepien et al., 2021). From a comparative point of view, mechanisms of cortex development at the level of repertoire of stem cells and proliferation strategies vary drastically even within closely related species of vertebrates and underly size and folding variation. The molecular roots of these differences are intensely studied by employing lissencephalic (harboring smooth brain) and gyrencephalic mammalians (showing superficial foldings and therefore expansion of the neocortex) (Lui et al., 2011; Dehay et al., 2015; Florio et al., 2015, 2016; Kalebic et al., 2019; Stepien et al., 2021). Furthermore, the presence of a homologous domain giving rise to the neocortex within the pallium territory in non-mammalian vertebrates is equally debated and little consensus exists to this day (Northcutt, 1995; Wullimann and Mueller, 2004; Medina and Abellán, 2009; Nieuwenhuys, 2009). Nevertheless, the basic core of orderly molecular and cellular processes governing anatomical and functional compartmentalization (bauplan) of the forebrain and fundamental principle of neurogenesis are conserved within the vertebrate lineage (Bachy et al., 2002; Puelles and Rubenstein, 2003; Wilson and Houart, 2004; Medina et al., 2005). Interestingly, specific cortical high-order cognitive abilities have also been mapped in teleosts (Portavella et al., 2002; Salas et al., 2003; von Trotha et al., 2014; Messina et al., 2022) and ancestral features can also be found in invertebrates (Williams et al., 2004; Cavallin et al., 2018).

The continuous identification of new genetic lesions affecting these different levels of neurogenesis and resulting in heterogeneous neurodevelopmental disorders with malformation of cortical development (MCD) highlights the importance of these processes during cortex formation. Improving our knowledge of the specific biology and genetics of pallium patterning, regionalization, precursor behavior and cell type specification and its variation on the main vertebrate theme can potentially impact our ability to model, understand and manage MCD. Indeed, these heterogeneous conditions can manifest with various degrees of microcephaly, lissencephaly and cortical dysplasia, originating from impaired proliferation, migration and/or white matter and circuits establishment. They are often associated with cognitive impairments and with at least 40% of non-treatable epileptic conditions (Guerrini and Dobyns, 2014; Barkovich et al., 2015; Represa, 2019). The clinical variability and genetic heterogeneity, as well as the poor knowledge on the underlying patho-mechanisms of the newly described conditions, often make these pediatric disorders not accurately managed. To this aim, non-primate mammalian systems such as rodents are undoubtedly providing an enormous help. Nevertheless, human-specific traits are often not successfully recapitulated in these models (especially related to folding formation) (Wong and Roper, 2016). On the other hand, more recently, the genetically accessible ferrets, gyrencephalic models with an expanded neocortex, are becoming popular to gain insights into neocortex expansion and to model specific traits which characterize human cortex development and disease (Hutsler et al., 2005; Martínez-Cerdeño et al., 2012; Florio et al., 2015, 2016; Fernández et al., 2016; Johnson et al., 2018; Kalebic et al., 2019; Pinson et al., 2019).

**FIGURE 1:**
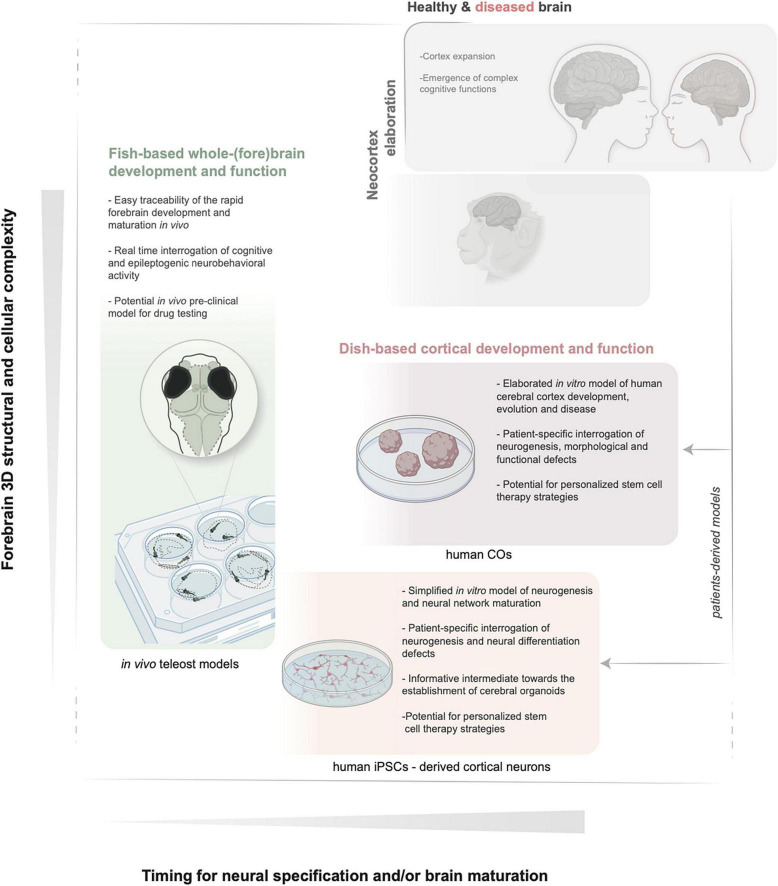
Schematic illustration of the key features of *in vivo* zebrafish and *in vitro* iPSCs and COs models. The relative increase in structural and functional complexity of the forebrain and the timing to obtain mature neuronal populations are indicated by the *y* and *x* axes, respectively. BioRender (BioRender.com) and Illustrator (Adobe) were used to generate the illustration. iPSCs, induced pluripotent stem cells; COs, cerebral organoids.

Nevertheless, given the current acceleration in the identification of candidate genetic lesions in previously undiagnosed MCD conditions, scientists are exploring alternative systems. These should (i) be more readily implementable than mammalian species in healthcare-oriented institutes, (ii) offer possibilities for fast candidate gene variants validation serving differential diagnosis, while (iii) parallelly allow the search of basic patho-mechanisms, and (iv) via methodologies which reduce the overall burden related to animals experimentation. To this aim, teleost fish can be advantageous (Figure 1 and Table 1). They represent the largest clade of vertebrates and, together with chick and mice, have long served as genetic and developmental models to study the principles of neural induction, and for the search of evolutionary paths to plasticity and adaptation of pallium formation and function within vertebrates (Salas et al., 2003; Menuet et al., 2007; Alié et al., 2018; Dunlap et al., 2019). Indeed, homologous neuronal ensembles, basal circuits and neurogenic modes within the pallium of teleost fish are being identified (Fotowat et al., 2019; Porter and Mueller, 2020). On this ground, and because of a number of factors determining its practical usefulness in developmental genetics and disease modeling, the small and transparent teleost fish zebrafish is effectively used. In many instances, the use of fish can replace less ethically acceptable methodologies required for brain analysis in mammalian embryos and young adults. Along these lines, more teleost models (such as medaka) are recently joining the forces and proving to be crucial to dissect multiple mechanisms of pallium expansion, vertebrate brain growth and rare brain diseases (Indrieri et al., 2013; Peluso et al., 2013; Kirchmaier et al., 2015; Dunlap et al., 2019; Coolen et al., 2020; Nakanishi et al., 2021).

**TABLE 1:** Glossary of the main terms employed in this review, including a brief description of the key models and processes discussed here.

Teleosts
**non-mammalian vertebrates** comprising the largest clade of ray-finned fish (Actinopterygii class). Different teleost species show various organisation of the brain (plasticity), which likely contributed to the different adaptation strategies to a variety of aquatic habitats, including fresh, sea, and brackish water. They can swim in shoals, an adaptive trait with antipredator purposes and show various reproductive strategies, but the majority reproduce via external fertilization; parental care is also observed in some species. *Zebrafish: freshwater fish belonging to Cyprinidae family. Largely employed in biomedical research (i.e. for human disease modeling) thanks to key advantages (refer to main text and table 3).*

**iPSCs (induced Pluripotent Stem Cells)**

**pluripotent stem cells obtained from somatic cells** (*i.e.*skin fibroblasts) following introduction of *Yamanaka transcription factors* and other small molecules (*e.g.* Forskolin or valproic acid) or by RNA-mediated reprogramming. iPSCs can be derived from adult tissues and directed towards different cell types (neurons, cardiomyocytes, pulmonary alveolar like-cells etc.), thus eliminating the need of embryonic cells to study proliferation and differentiation.

**Embryoid bodies (EBs)**

***in vitro* 3D cell aggregates derived from embryonic stem cells** (ESC), promoted by spontaneous adhesion between the cells and potentially differentiating into the three embryonic germ layers (endo-, meso-, ecto-derm).

**Organoids**

**3D self-organized tissue cultures** grown *in vitro* from stem cells (*i.e.*iPSCs and ESC) by simulating the multi-layer tissue formation and features of the original organ *in vivo* with self-renewal and differentiation capacities. Different strategies make it possible to obtain organoids of various types that model different brain regions, the retina, the kidney, organs from the gastrointestinal tract, etc. They can be obtained from various species (including humans) and can be employed in comparative developmental biology, disease modeling, and as pre-clinical drug testing. *Cerebral organoids (COs): a type of organoid containing cell types and cytoarchitecture that resembles the brain tissue, displaying various brain region identities (e.g. cortical layers) and cell types (e.g. outer radial glial stem cells, oRG and differentiated neurons). COs are being extensively used to study principles of mammalian, primate and human brain development and evolution as well as neurodevelopmental and neurodegeneration diseases.*

**Forebrain**

The anterior-most division of the brain, originated from the developing evaginating prosencephalon, followed by midbrain and hindbrain more posteriorly. The forebrain is divided into the **telencephalon** (with pallium and subpallium), and the **diencephalon** (which includes the thalamic region, pineal, hypothalamus, and habenula). In mammals, the telencephalon forms the characteristic cerebral cortex and the six-layered neocortex in the dorsal most of the pallium. Gyrencephalic mammals (such as ferrets and humans) have multiple foldings of the cortex which spatially expand the surface, serving circuits connectivity. The circuits derived from this region are responsible for high-order information processing, cognitive and associative functions, including modulation and control of the motor output. The forebrain in teleosts develops in the anterior-most part of the embryo by a different mechanism as compared to mammals (eversion). This results in a morphologically distinct topology, but conservation of the basic forebrain subdivisions is observed (with recognizable pallium and subpallium regions and derivatives), no six-layer cortex exists in this group of vertebrates, but functional homologous regions have been described.

**Neurogenesis and cell division during corticogenesis**

A process by which undifferentiated neural progenitor cells generate mature and functional neurons through I) the induction of cell division which enlarges the progenitor pool, II) the specification to committed neuronal progenitors and III) the differentiation to post-mitotic neurons and glial cells. In the mammalian developing cortex at the level of the ventricular zone apical and basal radial glia cells (aRG and bRG) are generated by neuroepithelial cells (NE) and represent the neuronal and glial precursor cells. Progenitor cells can divide by **symmetric cells division**, forming two progenitor cells from one. Neurons can be produced directly by a progenitor cell via **asymmetric cell division** producing one stem cell and a neuron or producing first an intermediate progenitor cell (IPCs, transit-amplifying cells) that can expand via one or two rounds of amplification before producing a neuron. Symmetric division can also be terminal and produce two neurons from one progenitor cell, thereby depleting the pool of proliferative precursors. RG cells are also found in teleosts. Postnatally, mammalian RG cells disappear giving rise to ependymal cells and astrocytes while teleost RG-like cells reach the quiescent state, conserving the ability to proliferate, self-renew and generate new neurons if necessary.

**Cortical neuronal migration**

An event in which post-mitotic cells within the developing cortex move away from the place of birth (ventricle) to reach their final position inside the layers and establish precise connections with other neurons. Neuronal migration contributing to cortex formation follows two main trajectories: I) radial and II) tangential. In the **radial migration**, occurring perpendicular to the surface of the cortex, cells born at the apical side lose the connections to both apical and basal surface and move along the RG to their target location and differentiate. In the **tangential migration**, cells move tangentially to the cortical plate, associate with RG, and move to their target location to undergo differentiation (i.e. interneurons). Cells moving by radial or tangential migration use different mechanisms for cell displacement (locomotion or somal translocation in radial migration while a peculiar saltatory movement with nuclear translocation is observed in tangential migration).

*Examples of specific models for teleosts and organoids are italicized.*

A different and complementary approach, quickly gaining popularity and reducing further the burden on animal models, is the establishment of induced pluripotent stem cells (iPSCs) directly from patients’ bioptic material and iPSCs-derived cerebral organoids (COs). The latter are becoming attractive *in vitro* alternatives in conjunction to the *in vivo* models to recapitulate species (i.e. human)-specific traits of early neurogenesis, differentiation, connectivity and even cytoarchitecture at small scale (Krefft et al., 2021; Zilova et al., 2021; Figure 1 and Table 1). Direct comparisons of COs development derived from different primates, mammals or other vertebrates are also becoming supportive tools for our understanding of the mechanisms underlying neocortex expansion (Li et al., 2017b; Kanton et al., 2019; Pollen et al., 2019). This review provides an updated overview of the key developmental steps and precursor cells that build the mammalian cortex. We next summarize representative examples of known genetic alterations impacting various molecular and cellular processes and leading to cortical malformations as the cause of intellectual disability and epilepsy. In the context of classifying and dissecting the causes of an increasing number of undiagnosed cortical malformations discovered via patients’ genomic screening, we next examine the possible value of the alternative and complementary fish and dish-based models to the *in vivo* mammalian systems. We discuss fruitful examples showing case their usefulness in contributing dissecting the fundamentals of neuronal precursors’ behavior and the underlying mechanisms in healthy and diseased brains. We specifically outline the limitations and challenges derived, for example, from the differences in pallium developmental processes and cell type repertoire (teleosts) or from the minimal tissue complexity and lack of whole-organismal context (organoids).

## Development and Disease of Pallium and Cortex Formation

Since the experiments of Spemann and Mangold in the early 20th century, developmental biologists used animal models to demonstrate that brain development is achieved by tightly controlled gene expression schemes that generate specific, recognizable local fingerprints. These events depend on the ability of cells in the early embryological fields to respond to varying doses of inducing and inhibiting molecules (morphogens) secreted by neighboring cells, which sequentially restrict the fate of each previously homogeneous domain, leading to patterning, regionalization, and cell type specification. These events step up the global brain architecture and its functional sub-specialization in different domains, and are globally conserved in evolution (Rallu et al., 2002; De Robertis, 2009). Understanding these processes has represented the ground research for flourishing comparative studies in developmental biology over the last decades that inspired various innovative experimental strategies. As a result, human genetic and brain diseases modeling *in vivo* have been improved and the breakthrough of organoids – including COs- is revolutionizing the study of the healthy and diseased mammalian brain.

## Patterning, Regionalization, and Precursor Cell Types

In vertebrate embryos, parallelly to the neural plate folding, signaling activated by retinoic acid, FGF, Wnt and Wnt inhibitors pattern the A-P (anterior-posterior) territories, while a fine-tuning of Bmp, Shh, and Wnt signals from axial mesodermal and non-neural ectodermal cells contribute to define instead the D-V (dorso-ventral) embryo axis (Wilson and Rubenstein, 2000; Houart et al., 2002; Rallu et al., 2002; Wilson and Houart, 2004). In this context, the establishment of the forebrain (Table 1) at the anterior bulge of the developing brain occurs already by the end of gastrulation, via a fine signaling balance of the different morphogens secreted from the organizer and the surrounding tissues (Rubenstein and Beachy, 1998; Stern, 2001; Wilson and Houart, 2004). A nested grid of highly conserved transcription factors is therefore generated and defines the boundaries between the different domains within the forebrain (Fernandez et al., 1998; Puelles et al., 2000). Key signaling events worth mentioning include the activation of Wnt inhibitors, e.g., Dickkopf-1 (Mukhopadhyay et al., 2001), Cerberus (Bouwmeester et al., 1996; Piccolo et al., 1999) and the secreted frizzled-related protein Sfrp3, (Kawano and Kypta, 2003) which counteract “caudalizing factors” and are essential for the vertebrate head formation. Crucial is also the modulatory activity of Fgf8 which globally sets the telencephalon aside from the neighboring diencephalon and contributes to its patterning (Shanmugalingam et al., 2000; Yamaguchi, 2001; Houart et al., 2002). This is practically orchestrated by *Bf-1* (Tao and Lai, 1992; Dou et al., 1999; Kitagawa et al., 2004), Emx, and Dlx genes (Fernandez et al., 1998; Puelles et al., 2000). The *Emx/Pax6* + dorsal pallium region of the telencephalon gives rise to the cortex, the medial pallium to the hippocampus and the ventral domain to the amygdala. On the ventral side of the telencephalon, the *Dlx* + subpallium is defined from where the basal ganglia and the hypothalamus originates. Posteriorly to the telencephalon, the diencephalic domain produces the thalamus and pretectum (Rubenstein et al., 1994; Rubenstein and Beachy, 1998; Puelles et al., 2000; Wilson and Rubenstein, 2000; Houart et al., 2002; Wilson and Houart, 2004; Cavodeassi and Houart, 2012).

The development and plasticity of the neocortex, originating from the dorsal pallium in mammals as a late evolutionary innovation (Striedter, 2005; Kelava et al., 2013; Namba et al., 2019) depends on the response of progenitor cells to the above mentioned modulatory signalings via a precise and stereotypical spatial-temporal logic of events. These ultimately control the correct proliferation rate of the initial pool of stem cells, the movement of the newborn neurons to their destination, their maturation in a number of different neuronal cell types and the establishment of their final connectivity pattern. From here, the resulting neural circuitries are distinctively wired, serving functional specialization for higher functions. Each of these events is especially critical to obtain the laminar arrangement of the mammalian pallium (Rakic, 2009a; Rakic et al., 2009b; Fietz et al., 2010; Salomoni and Calegari, 2010; Taverna et al., 2014; Subramanian et al., 2020), ultimately formed by circa 80% of excitatory neurons and the rest of inhibitory interneurons moving from the ventral telencephalon (Parnavelas et al., 2000; Lodato and Arlotta, 2015; Laclef and Métin, 2018). Briefly, in the region lining on the brain ventricle of the developing mammalian cortex (ventricular zone, VZ) the founder neuroepithelial cells with their characteristic apico-basal polarity along the radial axis undergo various rounds of symmetric cell division (“proliferative division,” Table 1). These events produce two new progenitor cells from one and therefore contribute to expanding the pool of progenitor cells. Apical and basal (or outer) (a, b RG cells, respectively) originated from these founder cells and expressing characteristic markers of glia cells (Götz and Huttner, 2005; Kriegstein and Alvarez-Buylla, 2009) maintain the apico-basal polarity and divide mainly by asymmetric cell division (“neurogenic division,” Table 1). This ensures the birth of new RG cells, serving self-renewal purposes (and thereby keeping the pool of stem cells), and differentiating neurons or various types of committed basal progenitors (BP), such as intermediate progenitor cells (IPCs). These cells migrate basally to the subventricular zone (SVZ) and produce neurons only after additional rounds of cell divisions (Noctor et al., 2001, 2004; Kriegstein and Götz, 2003; Haubensak et al., 2004; Kriegstein and Alvarez-Buylla, 2009; Uzquiano et al., 2018; Table 1). A great deal of variability exists across different mammalian species with respect to the fine details regarding the arsenal of progenitors’ cells, their morphology, and behavior, as well as the proliferation potential and the neurogenesis length (Betizeau et al., 2013; Kalebic et al., 2018, 2019; Stepien et al., 2020, 2021).

Importantly, the developing cortex of lissencephalic models, such as mice, have little bRG cells which divide both symmetrically and asymmetrically (Wang et al., 2011) and are instead mostly populated by IPCs-type of cells which have limited proliferative potential (Haubensak et al., 2004; Noctor et al., 2004). On the other hand, gyrencephalic animals like humans have evolved mechanisms to generate foldings which increase the overall surface and therefore host more neural cells in a limited space (Zilles et al., 2013). In these animals, the BP of the SVZ can sustain multiple rounds of divisions to generate other precursor cells before dividing into neurons (Götz and Huttner, 2005; Lui et al., 2011; Florio and Huttner, 2014). Indeed, the increase in the number of the BP outer RG cells (oRG) originating from aRG, and their proliferative potential has been proposed as a contributor for the increase in cortex size (Nonaka-Kinoshita et al., 2013; Fernández et al., 2016), but it does not seem to be sufficient, given the presence of lissencephalic animals with a high number of BP (Kelava et al., 2013; Stepien et al., 2021). Seminal works in the past years have demonstrated that a variety of morphology exists among BP cells even in the same species and that this instead might be a key factor determining neocortical expansion (Betizeau et al., 2013; Kalebic et al., 2019). The different BP morphotypes can vary from cells with monopolar to multipolar processes and it appears as if the increase in the number of processes boosts the proliferative capability, most likely by making the BP cells more available to external signaling. This mechanism might represent one of the major cellular features deciding cortex size expansion and neocortex appearance (Lui et al., 2011; Kalebic et al., 2019; Subramanian et al., 2020). Sustained gliogenesis in the outer SVZ has been implicated in the expansion of the neuropile, and therefore, the neocortex convolutions in primates (Rash et al., 2019). Of note, the genetic bases of such peculiarity of precursor cell types for neocortex expansion are mapped using gyrencephalic models (Florio et al., 2015, 2016; Cárdenas and Borrell, 2020; Heide et al., 2020; Stepien et al., 2021). At the signaling level, a number of pro-proliferative molecules from -and interacting with-the ECM niche of the progenitor pool, likely involving Pi3K-AkT, mTOR, and MAPK signaling pathways, is being recognized as a crucial trigger for the distinctive proliferative capacity of BP cells as well as for their positioning in the developing cortex (Sheppard and Pearlman, 1997; Fietz et al., 2012; Moreno-Layseca and Streuli, 2014; Cavallin et al., 2018; Heinzen et al., 2018; Long et al., 2018; Kalebic et al., 2019; Subramanian et al., 2020; Kyrousi et al., 2021).

Next, the basal projections from progenitor cells allow newborn neurons and glia produced by asymmetric division to migrate radially to the destination in the cortical plate (CP) by somal translocation or active locomotion (Sheppard and Pearlman, 1997; Nadarajah et al., 2001). The first migrating neurons populate the deep layer of the forming cortex, while newborn ones are located more superficially (Rakic, 1974; Buchsbaum and Cappello, 2019). Again, fine signaling is required for the different phases of migration, with Reelin being the most relevant to control different phases of this migratory activity (Trommsdorff et al., 1999; Kuo et al., 2005; Hirota and Nakajima, 2017). To complete cortex formation, inhibitory interneurons from the ventral telencephalon (the transient ganglionic eminences in humans) migrate long-range moving tangentially to the RG fibers to reach their final correct position and connect with other neurons in the CP (Lavdas et al., 1999; Bellion et al., 2005; Miyoshi et al., 2010; Faux et al., 2012; Barber and Pierani, 2016; Table 1). Their peculiar saltatory migration behavior, characterized by pauses in between fast periods of movement, is obtained via microtubules-dependent nuclear translocation (Bellion et al., 2005; Silva et al., 2018). This mechanism seems to be essential to control the stream of migratory cells populating the cortex and to even influence neurogenesis in the cortex (Silva et al., 2018). Lastly, cortical neurogenesis is nearly completed when gliogenesis starts, the remaining RG cells differentiate into astroglia, and the pool of progenitors is extinguished (Noctor et al., 2008; Ohtsuka and Kageyama, 2019).

## Malformations of Cortical Development (Mcd) Caused by Genetic Lesions Affecting Precursor Cells’ Behavior

The high complexity and specialization of the molecular and cellular events governing neocortex development and function in space and time make them particularly vulnerable to genetic alterations. A large group of malformations of cortical development (MCD) has been described which are characterized by various defects at the level of brain size (decreased or increased), final position of neurons, and formation of cortical layers and foldings (Toi et al., 2009; Guarnieri et al., 2018; Romero et al., 2018; Severino et al., 2020; Subramanian et al., 2020). Different conditions have been classically categorized based on the underlying perturbed cellular event, namely: alteration in the proliferative state of the precursors (a), migratory defects (b), or neural connectivity and circuits formations (c) (Table 2). For instance, microcephaly (reduced brain size) is mainly associated with unbalanced proliferation and/or apoptosis of the neuronal progenitors. Aberrant migratory behavior across developing cortical layers results in ectopic neurons located in the periventricular and subcortical region, disorganization and loss of cortical folding or lamination lead to gyrification defects and result in various heterotopias, polymicrogyria, lissencephaly and dysplasia (Guerrini and Dobyns, 2014). The latest acceleration in the use of genomic sequencing in undiagnosed patients reveals high heterogeneity in the genetic and cellular mechanisms of MCD, which often show shared cellular and morphological features, challenging the boundaries of the original definitions. Deepening our knowledge of the underlying mechanisms of the newly described conditions is particularly relevant to improving the functional classification of these disorders often associated with severe cognitive impairments (Guerrini and Dobyns, 2014) and untreatable epilepsy (Represa, 2019). Many disease-causing mutations have already been identified, which affect the function of key proteins involved in cytoskeleton, chromosomes arrangement machinery, organelle stability and bio-trafficking (Table 2). The fine spatial and temporal balance of these events in the developing pallium controls neurogenesis, migration, and connectivity. Of note, the sustained activity of signaling pathways involved is relevant for cortical malformations (Hong et al., 2000; Hevner, 2015; Jansen et al., 2015; Ossola and Kalebic, 2021; Table 2). We summarize the main features and known alterations of a subset of these conditions. For a more detailed classification, please refer to Table 2 and (Woods, 2004; Guerrini and Parrini, 2010; Barkovich et al., 2015; Watrin et al., 2015; Passemard et al., 2017; Buchsbaum and Cappello, 2019).

**TABLE 2:** MCD classification and representative examples of MCD-causing genes as well as IPSCs, COs and zebrafish models: the table summarizes the clinical classification and the disease mechanisms underlying different forms of MCD.

	Known genetic alterations	Representative zebrafish, IPSCs-derived neurons and COs models to study mechanisms underlying MCD
**Impaired neurogenesis and neural differentiation**		

***Primary microcephaly (congenital):*** Reduced intracranial brain volume (OFC < -2DS) present at birth		
***Secondary microcephaly:*** Reduced intracranial brain volume occurring postnatally (Woods, 2004; Subramanian et al., 2020)		

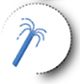 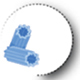	**Diseases associated with** **centrioles and tubulinopathies**^p^ *STIL* (#181590) *CENPJ* (CPAP, #608393) *CENPF* (# 600236) *ASPM (# 605481*) *TUBGCP5* (#608147) *TUBB5* (#615771) *KIF14* (#617914) *MCPH2* (*WDR62* accompanied with polymicrogyria and grey matter heterotopia, #604317) *MCPH3* (*CDK5RAP2*, #604804) *MCPH4 (CASC5*, #604321) *TBCD* (#617193) *TBCE* (#617207)	• ***MO-mediated cenpf KD in zebrafish embryos*** (Waters et al., 2015): increased embryo mortality and possible hydrocephalus, laterality defects due to cilia morphological alterations • ***CPAP-deficient human iPSCs and COs*** (Gabriel et al., 2016): cells from patients with Seckel syndrome show delayed cilia disassembly and thereby delayed cell cycle entry (G1-S) resulting in diminished RG cells and increased number of neuronal cells linked to premature neuronal differentiation. At the apical surface, the COs model shows an increased number of apical RG cells with altered cleavage plane (perpendicular to ventricular surface), indicating also an increased transition towards differentiation. Brain regions are smaller with a larger ventricle. • ***ASPM deficient human iPSCs and COs*** (Li et al., 2017a): impaired rosettes formation in neuronal progenitors derived from *ASPM* deficient patients-derived iPSCs. Organoids show loss of lumen structure, reduction of the number of ventral and outer radial glial cells and of mature neurons with dysfunctional calcium activity patterns • ***MO-mediated aspm KD in zebrafish embryos*** (Kim et al., 2011a): reduced head size, neuroectodermal cells show cell cycle arrest in metaphase stage, increased apoptosis is observed • ***kif14 KO and MO-mediated KD in zebrafish embryos*** (Reilly et al., 2019): increased embryo mortality, microcephaly, increased number of mitotic cells in the nervous system, impaired ciliogenesis • ***CDK5RAP2 deficient human COs*** (Lancaster et al., 2013): premature neuron differentiation at the expense of progenitor cells induced by a defective RG spindle orientation in patient-derived COs • ***WDR62 deficient human iPSCs and COs*** (Zhang et al., 2019): reduced NPCs proliferation, depletion of NPCs due to altered mitosis, survival, and balance between symmetric/asymmetric cell division with increased cell differentiation, size reduction observed also in COs with impaired cilia morphology (longer cilia and delayed cilia disassembly) • ***casc5 KO zebrafish embryos*** (Duerinckx et al., 2020): reduced head size • ***MO-mediated aspm and wdr62 KD in zebrafish embryos and stil*^cz65^* mutants*** (Novorol et al., 2013): reduced head size, failure to progress through prometaphase and increased apoptosis of retinal neuroepithelial cells • ***MO-mediated tbcd KD in zebrafish embryos*** (Pode-Shakked et al., 2017): microcephaly, reduced brain density and hydrocephalus
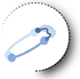	**Condensinopathies** *^p^* *NCAPD2* (#617983) *NCAPH* (#617985) *NCAPD3* (#617984) *NCAPG2* (#618460) *DONSON* (#617604, #251230)	• ***MO-mediated ncapg2 KD and KO in zebrafish embryos*** (Khan et al., 2019): microcephaly, altered mitotic progression of NPCs and increased apoptosis in the brain
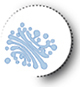	**Trafficking-related disorders** (e.g. Golgipathies) *ARF3* (Fasano et al., 2021)^p^ *ARFGEF2* (with periventricular heterotopia; #608097)*^s^* WDR81 (with lissencephaly; Cavallin et al., 2017)*^p^* *TRAPPC2L, TRAPPC6B, TRAPPC9, TRAPPC12* (#*618331, 617862, 613192, 617669)^s^* *COPB2* (#617800)^p^ *RAB18*(OMIM # 614222)*^s^*	• ***zebrafish embryos overexpressing ARF3 mutant proteins*** (Fasano et al., 2021)*:* microcephaly, fragmented Golgi and reduced cell protrusions and migration in early embryonic stem cells • ***MO-mediated trappc6b KD in zebrafish embryos*** (Marin-Valencia et al., 2018): reduced head size, increased apoptosis in the brain, increased susceptibility to seizures and neuronal hyperexcitability • ***MO-mediated rab18 KD in zebrafish embryos*** (Bem et al., 2011): microcephaly and developmental delay, reduced eye size and, delayed retinal development with abnormal retinal lamination
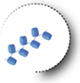	**Chromatin remodeling and** **DNA-RNA dynamics** *MECP2 (#312750) Rett syndrome* *KMT2A* (#605130)*^s^* *NACC1* (#617393)*^s^* *TLK2* (#618050)*^s^* *CHAMP1* (#616579)*^s^* *ARX* (#308350)^p^ *MCPH1* (#251200)^p^ *NARS1* (#619091, #619092)^p^ *VARS1* (#617802)^p^ *QARS1* (#615760)^p^	• ***NARS1 deficient human iPSCs and COs*** (Wang et al., 2020): reduced neural precursor cells in induced iPSCs and poorly organized and irregular-shaped radial glia cells with cell cycle defects • ***vars KO zebrafish embryos*** (Siekierska et al., 2019): microcephaly, increased apoptosis in the brain, increased susceptibility to seizures and neuronal hyperexcitability • ***qars KO zebrafish embryos*** (Zhang et al., 2014): microcephaly and increased apoptosis in the brain
	**Kinasopathies and others** *DYRK1A* (#614104)*^s^* *PTEN* (Dhaliwal et al., 2021)^p^ *PRUNE1* (#617481)^p^ *SLC25A19* (#607196)*^p^* *ASNS* (#615574)*^p^* *BBOX1* (Rashidi-Nezhad et al., 2014) *MFSD2A* (#616486) *PPP1R15B (#616817)*	• ***dyrk1aa KO zebrafish embryos*** (Kim et al., 2017): microcephaly, increased apoptosis in the brain, anxiety behavior and impaired social skills in adult fish • ***human COs overexpressing mutant PTEN*** (Dhaliwal et al., 2021): reduced size due to impaired neural precursor proliferation and premature neuronal differentiation mediated by a decrease in AKT activation • ***MO-mediated mfsd2a KD in zebrafish embryos*** (Guemez-Gamboa et al., 2015): early postnatal lethality and microcephaly with brain-blood barrier disruption

**Defective neuronal migration and connectivity**		

***Lissencephaly^type I^*:** simplification or absence of normal cortical convolutions in the cerebral cortex, often accompanied by secondary microcephaly (microlissencephaly) (Di Donato et al., 2017; Subramanian et al., 2020; Koenig et al., 2021)		
***Cobblestone lissencephaly ^type II^* :** global disorganization of cerebral organogenesis with an uneven cortical surface and a cobblestone appearance as well as demyelination (Devisme et al., 2012; Subramanian et al., 2020; Koenig et al., 2021)		

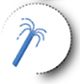	***Tubulinopathies*** *^type I^* *TUBA1A, TUBA3* (associated to PMG, #611603) *TUBB2B* (associated to PMG, #610031) *KATNB*1 (associated to microcephaly, #616212) *LIS1* (#607432, #247200) *Isolated lissencephaly and Miller-Dieker* syndrome (MDS), often accompanied with subcortical band heterotopia and/or PMG *DCX* (#300067) *X-linked* *lissencephaly and double cortex syndrome* (subcortical band heterotopia, see below) *ARX*#300215 - *X-linked* *lissencephaly with agenesis of* *corpus callosum (XLAG)*	• ***TUBA1A deficient human iPSCs*** (Bamba et al., 2016): inhibition of neurit eextension in young neurons • ***MO-mediated katnb1 KD in zebrafish embryos*** (Mishra-Gorur et al., 2014): microcephaly with decreased midbrain size • ***MDS-induced human iPSCs and COs*** (Bershteyn et al., 2017): reduced organoid size, increased apoptosis and horizontal cell division with vertical spindle orientation of NPCs, prolonged mitosis of oRGCs and neuronal migratory defects in patient-derived COs • ***DCX deficient human iPSCs*** (Shahsavani et al., 2018): impaired migration and prolonged proliferation of neural stem cells, defective neuronal differentiation and neurite extension • ***arx KO zebrafish embryos*** (Griffin et al., 2021): reduction in the forebrain interneuron density, hypoactivity associated with unprovoked seizures identified by electrophysiology
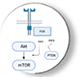	***Reelinopathies*** *^type I^* *RELN* (#257320) *VLDLR* (#224050, LDLR-Associated Cerebellar Hypoplasia associated with mild signs of lissencephaly) *DAB1* (Smits et al., 2021)	• ***reln, vldlr and dab1a mutant zebrafish embryos*** (Nimura et al., 2019): aberrant positioning of Purkinje cells (*reln, vldlr* and *dab1a*), eurydendroid cells (projection neurons), and Bergmann glial cells in the cerebellum (*reln*) accompanied to ectopic neurons in the tectum (*reln, vldlr*, and *dab1a*)
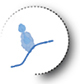	**Muscular dystrophy-causing dystroglycanopathies** (*i.e., Walker Warburg syndrome*, *WWS*) associated with brain, eye/retinal defects, lissencephaly and PMG/agyria)*^type II^* *POMGnT1* (#253280) *POMT1* (#236670) *POMT2* (#613150) *POMK* (#615249) *LARGE1* (#613154) *FKRP* (#613153) *FKTN* (#253800) *B3GNT1* (#615287) *B3GALNT2* (#615181) *ISPD* (CRPPA, #614643) *TMEM5 (# 6150741)* TMTC3 (#617255)	• ***pomgnt1*^sny7^* and pomgnt1*^sny47^* mutant zebrafish embryos*** (Liu et al., 2020): retinal photoreceptor (PR) degeneration associated with impaired O-mannosyl glycosylation, loss of matriglycan and retention of EYS-enriched secretory vesicles (synaptotagmin-1-positive) in the PR outer nuclear layer • ***MO-mediated b3gnt1, b3galnt2, fktn and fkrp KD in zebrafish embryos***: muscle defects (U-shaped somites) with sarcolemma disruption and degeneration associated with reduced glycosylation of αDG (Buysse et al., 2013, *b3gnt1*), ER stress and loss of dystroglycan– ligand interactions (Lin et al., 2011) (*fktn* and *fkrp*) or reduced mobility, hydrocephalus and mild retinal degeneration (Stevens et al., 2013) • ***MO-mediated ispd KD in zebrafish embryos*** (Roscioli et al., 2012): WWS model showing hydrocephalus, reduced eye size, muscle defects and degeneration associated with hypoglycosylated αDG

***Polymicrogyria (PMG):*** excessive number of abnormally small cerebral gyri (Stutterd et al., 1993)		

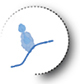	**PMG - causing dystroglycanopathies/laminopathies** *GPR56* (*ADGRG1*, associated with cerebellar and white matter abnormalities; #606854, #615752) *LAMA2* (#607855) *LAMB1 (#615191)* *LAMB2* (#615191) *LAMC3* (#614115) ***Other cell cycle-related lissencephaly*** *^type I^* *NDE1* (#614019) *CDK5* (#123831)	• ***gpr56 KO zebrafish embryos*** (Ackerman et al., 2015): significant reduction of mature oligodendrocytes’ number and myelinated axons due to decreased proliferation of oligodendrocyte precursor cells • ***lama2 mutant zebrafish embryos*** (Gupta et al., 2012): muscle degeneration, brain size reduction with clumped cells, eye size reduction with compressed cellular layers, associated to reduced ECM
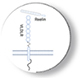	***PMG-causing mTORpathies*** *AKT3* (associated with FD #615937) *CCND2* (#615938) *MTOR* (#616638) *PI4KA* (#616531) *PIK3CA* (#602501) *PIK3R2* (#603387) *PTEN* (Shao et al., 2020)	• ***MO-mediated pi4ka KD in zebrafish embryos*** (Ma et al., 2009): decreased cell proliferation and increased apoptosis throughout the embryo (including the brain) • ***PTEN-deficient human COs*** (Li et al., 2017b): increased NPCs proliferation and pool expansion, increased folding and size of developing COs via PTEN-AKT signaling, associated with transient delay in neuronal differentiation (not observed in mouse *PTEN-*deficient COs)
	**Other PMG-causing disorders** 22q11.2 deletion (#611867) 1p36 deletion (#607872) *COL18A1* (#267750) *COL4A1, COL4A2* (Cavallin et al., 2018) *FIG4* (#612691) *OCLN* (#251290) *GPSM2* (#604213) *PAX6* (#106210) *RTTN* (#614833) *SNAP29* (#609528)	• ***fig4a*^cq35^* mutant zebrafish embryos*** (Bao et al., 2021): increased vacuolation in various tissues, including brain, associated to lysosomal storage defects and containing autophagic intermediates • ***MO-mediated pax6 KD in zebrafish embryos*** (Coutinho et al., 2011): small central nervous system and reduced eyes size, impaired proliferation and differentiation within the nervous system • ***PAX6 deficient human COs*** (Xu et al., 2021): impaired telencephalon differentiation dependent upon altered interaction with LncRNA PAUPAR and the histone methyltransferase NSD1 which regulate H3K36 methylation and expression of target genes involved in cortical differentiation • ***snap29 mutant zebrafish embryos*** (Mastrodonato et al., 2019): increased apoptosis during early stages, which is associated with accumulation of autophagy markers and aberrant multilamellar organelles. Excessive neuronal branching and locomotor impairment

***Grey matter heterotopia (subependymal/subcortical and band):*** ectopic positioning of neurons during cortex development (with formation of ectopic nodules) (Watrin et al., 2015; Subramanian et al., 2020)		

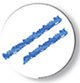	**Actin-cytoskeleton and cell-adhesion disorders** *FLNA* (#300049) *FAT4* (#615546) *DCHS* (#607829, #601390)	• ***MO-mediated flna KD in zebrafish embryos*** (Adams et al., 2012): Meckel–Gruber syndrome-like phenotype (ciliopathy), with pronephric cysts, hydrocephalus and notochord abnormalities • ***FAT4 and DCHS deficient human iPSCs and COs*** (Klaus et al., 2019): patients-derived and isogenic knock-out lines. Altered neuronal morphology and migration abilities, resulting in neurons accumulating in the VZ compartment
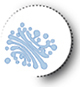	**Trafficking-related disorders** (e.g. Golgipathies) *ARF1* (# 618185)	***zebrafish embryos overexpressing ARF1 mutant proteins*** (Carvajal-Gonzalez et al., 2015): impaired axial morphogenesis, embryo elongation and notochord formation likely associated with defective stem cell polarity
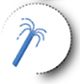	**Tubulinopathies** *EML1* (#600348) *TUBG1*(#615412) *DYNC1H1*(#614563) *KIF5C* (#615282) *KIF2A* (#615411) MAP1B (associated with PMG; #618918)	• ***kif2a KO zebrafish embryos*** (Partoens et al., 2021): microcephaly, reduced NPCs proliferation, increased apoptosis. Evidence of susceptibility to seizures and cognitive impairments • ***MO-mediated dync1h1 KD in zebrafish embryos and dync1h1*^mw20^*KO mutants*** (Insinna et al., 2010): defective morphogenesis of outer segment in photoreceptors, associated with cell polarity and organelle positioning defects
	**Others** *NEDD4L* (#617201) *LGALS3BP* (Kyrousi et al., 2021; associated with periventricular nodular heterotopia and microcephaly) *ECE2* (Buchsbaum et al., 2020)	• ***LGALS3BP deficient COs and human fetal brain*** (Kyrousi et al., 2021): delayed growth of iPSCs-derived COs, altered distribution of NPCs, found mostly in mitosis and accumulated at the apical surface in proximity to the ventricles, increased number of ectopic newborn neurons in the VZ compartment in both COs and human fetal brain and altered composition of secreted proteins resulting in a loosening of apical belt in COs • ***ECE2-deficient iPSCs and COs*** (Buchsbaum et al., 2020): increase of differentiated neurons ectopically located in the VZ, slow migration, increased tortuosity, and pausing time of young migrating neurons. Increased thickness of F-actin-enriched adherent junctions and altered neuroepithelium with loss of apical junctions, reduction/disorganization of stabilized microtubules in germinal zones showing altered apico-basal polarity of RG cells, due to altered intracellular matrix composition and integrity as revealed by whole-proteome analysis in ECE2-deficient COs

***Focal cortical dysplasia (FD):*** disorganized cortical lamination (FD type Ia, Ib, IIa, IIb, III). Often associated with polymicrogyria (Najm et al., 2018; Subramanian et al., 2020) Tuberous sclerosis (TS): cerebral cortical tubers and subependymal nodules with dysmorphic, disorganized neurons and reactive glia in the cortex, often linked to epileptogenesis (Subramanian et al., 2020; Zimmer et al., 2020)		

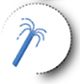	**Tubulinopathies** *TUBB2B* (associated to PMG, FD # 610031) *TUBB3* (associated with PMG and microlissencephaly, FD #614039)	
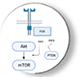	**FD-causing mTORpathies** *TSC1* and *TSC2* (LoF somatic mutations causing FD, #607341; *RHEB* (Zhao et al., 2019) *MED16* (Zhao et al., 2019) AKT3 (Alcantara et al., 2017) PIK3CA (Jansen et al., 2015) PIK3R2 (associated with PMG) (Terrone et al., 2016) DEPDC5 (second-hit mosaic mutations in the gene cause FD, #604364) **TS-causing mTORpathies** Germline biallelic LoF mutations causing TS, #613254, # 191100) **Others** SLC35A2 (Bonduelle et al., 2021)	• ***tsc2*^vu242/vu242^*zebrafish mutants*** (Kim et al., 2011b; Kedra et al., 2020): defective axons fasciculation in migrating neurons, thinner anterior commissures in the telencephalon, extensive gray and white matter (WM) disorganization with ectopically positioned cells and WM dysconnectivity resulting from aberrant axon elongation. Reduced locomotor response to a light-dark stimulus and increased anxiety-like behavior and epileptogenesis (Kedra et al., 2020) • **TSC1 and TSC2 deficient human cortical spheroids** (Blair et al., 2018): induced mTORC1 signaling inducing a bias towards gliogenesis at the expenses of neurogenesis. Reduced expression of neural markers and increased expression of glia markers • ***rheb zebrafish mutants*** (Reijnders et al., 2017): increased head size, defective neuronal migration and increased soma size, susceptibility to seizures

Relevant cellular and molecular insights into the mechanisms of disease from representative in vivo zebrafish and human IPSCs and COs models are shown in the right column.

^p^ = primary,

^s^ = secondary, iPSCs = induced pluripotent stem cells, PSCs = pluripotent stem cells, ESC = embryonic stem cells, COs = cerberal organoids, KD = knock-down, KO = knock-out, MO = morpholino, NPCs = neuronal progenitor cells, αDG = alpha-dystroglycan, ECM = extracellular matrix, VZ = ventricular zone.

## Microcephaly

Microcephaly is a rare clinical trait defined by the reduced head and brain size, with an occipital-frontal circumference often below the 3rd percentile. It can occur at birth (primary), or postnatally (secondary), as an isolated condition or in combination with other clinical features, i.e., developmental delay, epilepsy, and CNS malformations (Abdel-Salam et al., 2000; Woods, 2004; Marino et al., 2020; Becerra-Solano et al., 2021), as well as intellectual disability (Marino et al., 2020). Aside from environmental factors and viral infections, genetic mutations represent the most frequent underlying cause (Table 2). Indeed, many mutations in conserved genes have been already identified, which negatively impact progenitors’ expansion as well as migration of newborn neurons. Proteins directly controlling microtubules dynamics (Chakraborti et al., 2016) (e.g., KIF14, TBCD) (Filges et al., 2014; Flex et al., 2016), centrosome biogenesis (e.g., STIL) and centrosome proteins regulating microtubules and spindle orientation (e.g., ASPM) (Darvish et al., 2010) are often involved. The involvement of factors controlling chromatin organization (e.g., MCPH1) (Darvish et al., 2010) as well as altered kinase activity (e.g., PTEN) (Dhaliwal et al., 2021) and abnormal substrate phosphorylation of phosphates (e.g., PPP1R15B, (Kernohan et al., 2015) have also been described. The resulting alteration of mitotic spindle formation and orientation, cell cycle, as well as proper chromosome segregation during mitosis often generates a shift between symmetric versus asymmetric cell division, depleting the pool of progenitor cells and causing apoptosis of early precursors. A different group of genes causing microcephaly and linked functionally to Golgi homeostasis and trafficking is also emerging. For instance, secondary microcephaly combined with periventricular heterotopia was linked to impaired Golgi assembly and trafficking affecting membrane proteins, proliferation and cell migration (e.g., ARFGEF2, Banne et al., 2013). Furthermore, fragmented Golgi has also been recently associated with a neurodevelopmental condition showing microcephaly and caused by mutations in the new disease-gene *ARF3*, encoding a small GTPase involved in post-Golgi trafficking (Fasano et al., 2021). More recently, loss of function of *WDR81* which impairs endosomal trafficking of pro-proliferative signal EGFR was functionally linked to microcephaly (Carpentieri et al., 2022).

## Cortical Malformations Mainly Derived From Migration Defects

Impaired migration of newborn neurons from the ventricle to the CP along the RG scaffold causes different forms of neuronal migration disorders (NMDs). This is a group of highly heterogeneous conditions, which can show mixed features, including microcephaly or other cerebral malformations (Sheen et al., 2006; Lui et al., 2011; Buchsbaum and Cappello, 2019; Table 2). Among NMDs, lissencephaly (also known as “smooth brain”) refers to a large number of heterogeneous conditions, mostly characterized by an aberrant and reduced cerebral folding, in combination with other cerebral malformations; while gray matter heterotopia specifically refers to abnormally located neuronal clusters (Watrin et al., 2015; Di Donato et al., 2017; Buchsbaum and Cappello, 2019; Buchsbaum et al., 2020; Koenig et al., 2021). Furthermore, neuronal migration errors (Guerrini and Parrini, 2010) or aberrant proliferation of glia precursors (Rash et al., 2019) also lead to polymicrogyria, characterized by architectural anomalies and cytological variations involving the number of cortical convolutions. Abnormal focal migration of the newborn neurons causing impaired cortical lamination has also been linked to focal cortical dysplasia, which is among the most frequent causes of refractory epilepsy in children (Tassi et al., 2002; Guerrini and Dobyns, 2014). As previously discussed, interneurons are essential for correct cortex formation and to develop healthy connectivity and signal transmission (André et al., 2010). Impaired behavior during their long migratory tour from the ventral telencephalon to the cortex and their arborization can cause a disorganized lamination with impaired circuits activity within the cortex, leading to epilepsy and cognitive dysfunction (Pancoast et al., 2005; Carabalona et al., 2012; Nakagawa et al., 2017; Buchsbaum and Cappello, 2019; Subramanian et al., 2020). Furthermore, studies exist that functionally correlate the alterations in interneurons’ location and connectivity patterns to seizures occurrence in cells derived from patients with focal cortical dysplasia (Calcagnotto et al., 2005; Cepeda et al., 2005; Subramanian et al., 2020).

At the molecular level, these conditions are associated with mutations in genes encoding proteins involved in microtubules dynamics and transport cytoskeleton-cell matrix interactions, e.g., *LIS1, DCX, KIF2A, TUB1A*, (Chakraborti et al., 2016), *MAP1B*, (Heinzen et al., 2018), *ARX, FLNA* (Parrini et al., 2006), *ARFGEF2*, (Sheen et al., 2004). Mutations in these genes hinder neuronal polarity and cause migratory impairment and unbalanced proliferative potential of the precursor cells (Carabalona et al., 2012; Buchsbaum and Cappello, 2019). In addition, impaired signaling, caused by mutations in key molecules of the mTOR, FGF and REELIN signaling and ECM components, can impair proliferation, migratory behavior or neurites formation during cortex development leading to severe forms of MCD. Among these, lissencephaly (Hong et al., 2000) and polymicrogyria besides characteristic forms of muscular dystrophy (Devisme et al., 2012) and polymicrogyria (Godfrey et al., 2007).

## A Search for Alternative Models to Assess Fundamental Mechanisms of Pallium Development and Disease: Do Teleost Fish and Organoids Qualify?

As discussed, new disease genes and candidate pathogenic variants involved in MCD are continuously described, which may underlie the poorly unexplored heterogeneity in the clinical, genetic, and mechanistic aspects of cortical malformations. Yet, our little understanding challenges the utility of the existing disease classification and the development of tailored therapies. Classically, mouse models have provided -and continue to provide- considerable experimental evidence into the fundamental mechanisms of forebrain development and cortex formation in health and disease (Buchsbaum and Cappello, 2019; Reinert et al., 2021). For instance, mutant studies in mice models have predicted the involvement of Reelin signaling in complex forms of cortical malformation before the actual discovery of patients harboring mutations in genes belonging to the signaling cascade (D’Arcangelo et al., 1995; Trommsdorff et al., 1999; Hong et al., 2000). On the other side, successful models for deciphering the mechanisms underlying differences in neocortex size are primates, and more recently ferrets, gyrencephalic mammals, which harbor an expanded neocortex likewise humans and for which *in utero* genetic manipulation of embryonic brain is now possible (Hutsler et al., 2005; Florio et al., 2015, 2016; Gilardi and Kalebic, 2021). These models are particularly beneficial for recapitulating specific patho/physiological traits of MCD, especially when addressing cortical growth and folding defects, which were not previously observed in mammals with smooth cortex such as mice (Masuda et al., 2015; Kalebic et al., 2019; Pinson et al., 2019).

The acceleration of large patients’ sequencing efforts made possible by advances and increased affordability of the NGS (next-generation sequencing) on one side and the consensus on minimizing the animal distress associated with the invasive nature of experimentation in these large animals, quest for possible alternative models. These might be used for fast and large-scale functional validation of variants of unknown impact. Ideally, valid model systems successfully complementing the established and emerging mammalian ones should: (i) be rapidly implementable and manageable in medical research institutes, (ii) bring to a fast validation of the candidate genetic variants, (iii) while reducing the reliance on invasive animal procedures and still (iv) allowing scientific and translational gains in terms of mechanistic understanding of the disease. In this sense, despite not harboring the multi-layered cortex and given the conservation of fundamental mechanisms, the arsenal of genetic, imaging, and behavioral tools, teleost fish (Figure 1 and Table 1) are actively being used. Many studies now provide a critical comparison of telencephalon development and the specific neurogenic programs in teleosts. This knowledge is beneficial for data interpretation, given the increased use of these models (especially zebrafish) for validating MCD-causing mutations and deciphering the underlying mechanism (Table 2).

Parallelly, a different *in vitro* approach is also gaining increasing popularity, which is the use of iPSCs and COs derived from patients’ fibroblasts (Figure 1 and Table 1). Despite many drawbacks associated with the *in vitro* nature of the system, these models are useful to directly assess certain mechanisms of human cortex neurogenesis in health and disease or to draw hypotheses that can help strategize *in vivo* experiments. This section examines the ground similarities and differences between teleost and mammalian pallium and the use of zebrafish, iPSCs and COs for brain development and disease, highlighting representative examples. We discuss the main benefits and limitations, which still need to be overcome to improve the translational value of these models.

## Teleosts: Understanding Conservation and Changes in Pallium Development

The genetic and molecular paradigms of the ground patterning and regionalization of the forebrain are conserved within various vertebrates (Puelles et al., 2000; Wilson and Houart, 2004; Medina et al., 2005; Murakami et al., 2005) and even in more distantly amniotes vertebrates, i.e., teleost fish (Heisenberg et al., 1996; Fernandez et al., 1998; Shinya et al., 2001; Puelles and Rubenstein, 2003; Kitagawa et al., 2004; Ganz et al., 2012). These processes have deep roots in evolution such that the molecular topography of a “paleopallium” can be traced back even to basal Bilateria (Medina and Abellán, 2009; Tomer et al., 2010).

Teleost fish models are a successful radiation of vertebrates and account for at least half of existing ones. They have populated developmental biology and neurobiology laboratories since the first studies on body plan formation and neural induction. These fish have been instrumental in establishing the first embryological events patterning the vertebrate telencephalon (e.g., see Houart et al., 1998; Kitagawa et al., 2004; Hashiguchi and Mullins, 2013), and complementing findings in mouse and other species. However, it should be noted that, conversely to mammals, teleost fish, show a “non-laminar” pallium structure that does not generate a *bona fide* cortex (Ito and Yamamoto, 2009). Instead of forming by invaginating cells as in mammals, the teleostean pallium domain is generated by an eversion process, whose underlying mechanisms are still under debate. This process ultimately generates a medial T-shaped ventricle and two lobes, in net contrast with the overall mature morphology of the mammalian counterpart (Wullimann et al., 1996; Wullimann and Mueller, 2004; Nieuwenhuys, 2009; Yamamoto, 2009). To this date, there is a lack of a solid conclusion on the existence of homologous forebrain and pallium sub-domains between teleosts and mammals. Hence, the teleostean pallium has long been considered rather “simple” because of these morphological differences and the uncertainty on assigning homology. This has precluded teleost models for a long time from further exploitation in neuroscience and brain disease modeling and limits the use and data interpretation of teleost fish in the field of MCD. Nevertheless, it is largely accepted by a wide community that, to a certain extend, an embryological origin, anatomical and functional homology exists between mammalian and teleost forebrain (Wullimann et al., 1996; Wullimann and Mueller, 2004; Nieuwenhuys, 2009; Yamamoto, 2009). Anatomical and functional components homologous to a number of pallial domains which originated from the dorsal portion of the telencephalon have already been proposed. In the course of the last decade, researchers have found in teleost fish regions homologous to the amygdala driving motivated behavior (von Trotha et al., 2014; Porter and Mueller, 2020), a domain resembling the hippocampus, which mediates memory and spatial information (Portavella et al., 2002; Salas et al., 2003; Rodríguez et al., 2021) and one responsible for cortex-type of high cognitive functions, such as logical and inference to determine social status (Grosenick et al., 2007) or numerosity tuning (Messina et al., 2022). On the other side, differences and plastic changes of the brain and pallium development at the level of morphology, connectivity and function exist across Bilateria and within vertebrates (Northcutt, 1995). One could say that the pallium itself underwent multiple events of sophistication and diversification in aquatic and land vertebrates, likely due to the elaboration of early stem cells independently from allometric relationships (Striedter, 2005; Noreikiene et al., 2015; Montgomery et al., 2016). Such phenotypic plasticity independently of other brain regions is manifest in teleosts, which results in considerable variations in the forebrain topology, neuroanatomy, and functional organization, likely underlying their ability to adapt to a variety of environments (Ito et al., 2007; Sylvester et al., 2013). Therefore, it should be mentioned that teleosts also represent an experimental advantage to showcase fundamental principles on how brain modularity and diversification within and across species is obtained. As shown indeed, this can occur by genetic changes (adaptation), which trigger mosaic responses in basal and shared neurogenic programs and thereby underly anatomical changes (Rétaux et al., 2013; Hall and Tropepe, 2020), as also demonstrated within mammals (Florio et al., 2015, 2016; Kalebic et al., 2018, 2019; Stepien et al., 2021). These changes can affect early patterning and sub-domain fate decision, therefore proportioning brain regions via directly modulating, the timing of local neurogenesis, cell cycle state and migratory behavior. Examples of the relevance of these shifts for forebrain plasticity in domain size and function were accumulated from comparative studies in teleosts. For instance, even small heterochronic and heterotopic variations in Shh and Fgf signaling doses can determine morphological and functional changes in the development of various forebrain domains in different teleosts. Namely, the expansion of Shh secreted from the midline was causatively linked to the expansion of the diencephalic domain (i.e., of the ventral hypothalamic region) in cave-dwelling cavefish as compared to the surface conspecifics adapted to surface. Of note, species differences also at the level of neuropeptidergic cell clusters (e.g., an increase in orexigenic NPY or Hcrt producing cells), controlling behavioral adaptations such as locomotion likely for foraging needs, were sustained in cave-adapted fish (Menuet et al., 2007; Alié et al., 2018). Again, evidence exists also in sand and rock-dwelling cichlids that similar variations in competing Shh and Wnt signaling expand or restrict the dorsal telencephalon (pallium). Specifically, an early function of Wnt inhibitors in development produces a larger pallium in sand dwellers fish as compared to the rock counterparts. In the latter on the other side expansion of Shh signaling and blocking Wnt inhibitors produce an extended subpallium. The potential modulatory effect of changes in these signalings on telencephalon development was further demonstrated by manipulating them in zebrafish (Rétaux et al., 2013; Sylvester et al., 2013).

When considering teleost fish as neurodevelopmental and disease models, one should also consider that the enrichment of progenitor cell types, their proliferative potential, the migratory routes of the newborn neurons resulting in the peculiar laminar organization of the neocortex are distinctive features of mammals, further elaborated in primates and in some gyrencephalic animals. Nevertheless, despite the differences discussed before, conservation of the founder precursor cells and the core mechanisms of neurogenesis exists in teleost telencephalon, as well as the functional competence derived from it (Portavella et al., 2002; Salas et al., 2003; Wilson and Houart, 2004; Nieuwenhuys, 2009; Rakic, 2009a; von Trotha et al., 2014; Porter and Mueller, 2020). Specifically, when it comes to the compendium of cell types, RG cells with proliferative potentials exist in various teleost’s brain regions, including the telencephalon. Debate exists on whether radial migration occurs during pallium development in these fish. Interestingly, (Mueller et al., 2011) described proliferative (Brdu +) cells migrating from the medial proliferative area of the dorsal telencephalon to the lateral and posterior area of the pial surface in developing zebrafish forebrain between 3 and 8 days post-fertilization. Similarly, photoconversion experiments in zebrafish provide evidence of a migratory cell population moving from the proliferative VZ of the developing pallium to the lateral wall during expansion of the telencephalic domain between 2 and 5 days of development (Folgueira et al., 2012). However, this behavior was not confirmed by later birth-dating experiments (Furlan et al., 2017); while findings of potentially migratory RG-like cells have been recently proposed also in cichlids (Mack et al., 2021). Noteworthily, conversely to mammalian species, teleost RG cells conserve the ability to proliferate and give rise to newborn neurons that integrate into existing circuits, in various neurogenic areas of the brain also in adulthood and in response to injury and disease. This feature defines the distinctive regenerative potential of the teleosts brain (Zupanc and Clint, 2003; Strobl-Mazzulla et al., 2010; Baumgart et al., 2012; Lange et al., 2020) and was also described for other non-mammalian vertebrates (García-Verdugo et al., 2002). Of note, the use of transgenic lines in zebrafish allowed also the *real time* visualization of migratory interneurons from the developing ventral to the dorsal telencephalon (Mione et al., 2008), resembling the saltatory migratory behavior observed in mammals and other vertebrates (Bellion et al., 2005; Laclef and Métin, 2018). In addition, other newborn neurons migrating out of the proliferative zones have been found in various sites of the adult zebrafish brain. Tangential migration of interneurons from ventral telencephalon was documented, which resembles rodent and tetrapods rostral migratory stream populating the anterior-most part of the telencephalon, the olfactory bulb. Altogether the available data seem to support a possible conserved mechanism of dual origin and migratory behaviors contributing to telencephalon cell types, and highlight zebrafish as an interesting model to study and visualize the mechanisms of these processes not invasively (Doetsch et al., 1997; Byrd and Brunjes, 2001; Grandel et al., 2006; Alvarez-Buylla et al., 2008; Mione et al., 2008; Ganz et al., 2012). Recent work demonstrates also the utility of alternative teleost models (such as killifish) besides zebrafish to dissect the impact of divergent and adaptive mechanisms at the level of precursor cells and neurogenesis and their function in sustaining pallium growth in vertebrates (Coolen et al., 2020).

### Zebrafish Model and Advantages

Teleost fish are successful vertebrate models for developmental neurogenetics to study mechanisms of vertebrate brain plasticity, development, and function, as well as human brain diseases. Zebrafish, the most famous member of this clade, is increasingly becoming part of interdisciplinary research efforts that aim to establish fast causal links between newly discovered gene variants in undiagnosed patients and heterogeneous MCD. Zebrafish models of different classes of MCD (i.e., tubulinopathies, golgipathies, and dystroglycanopathies) affecting cortex formation in humans have already contributed to our understanding of pathogenic variants and mechanisms involved in the disease (Table 2).

Numerous are the features and benefits that favor the use of zebrafish in neurodevelopmental biology and disease modeling (listed in Table 3). In detail, this fresh water fish is a small laboratory pet, originally collected from Asia and already employed early on in large mutagenesis screens to map gene function during vertebrate development (Amsterdam et al., 1999), similarly to the other famous teleost from rice fields, medaka (Loosli et al., 2000). Reaching only few mm in adulthood, zebrafish can be relatively easy to handle. It usually adapts well to laboratory raring conditions, for which high-standard - and continuously updating guidelines- are available (Aleström et al., 2020). Conversely to rodents and other large mammalian models, fertilized zebrafish eggs develop fast and externally from the mother. This allows to collect and analyze embryos without animal suffering. Furthermore, given the fast embryogenesis, one can follow the entire process of gastrulation, embryo/brain patterning and regionalization from anterior to posterior within hours after the fertilization event, from 4 to 5 hours up to 5–6 days of development. At this stage small fish reach the status of an independent feeding larva with a fully functional nervous system. In addition, under the right housing and raring conditions, these fish become sexually mature and can reproduce in less than 6 months generating large offspring (between 200 and 300 embryos per clutch). Compared to other mammalian systems, these features are extremely useful when generating transgenic and mutant lines. A stable line in zebrafish can be produced in less than a year. Such high fecundity also allows a sufficient sample size for statistical analysis, again minimizing additional adults’ use for breeding. Compared to mice models, this feature enables easier screening and selection of the animals carrying the desired mutant and transgenic alleles and reduces line generation and maintenance costs. Transient transgenesis to mark a few cell types and dynamics are also widely used in zebrafish, which allows analysis of transgenic animals even within few days from the microinjections performed to obtain the genetic modification (Kawakami, 2005).

**TABLE 3:** Key features and main limitations of zebrafish, iPSCs and organoids.

Key general features	Main limitations
***Zebrafish***	

• **Fast reproduction time and high fecundity** ensures a large number of embryos per experiment (including drug screening), reduces the possible burden associated with breeding procedures and facilitates the selection of the desired allele while establishing a transgenic and mutant line • **External egg fertilization** eliminates the need of invasive procedures for embryos collection or genetic manipulation necessary in mammalian models or other larger animals • **Transparent embryos** and young larvae develop fast, which are ideal for non-invasive brain development (that can be followed already at 10 hours after fertilization) and functional brain imaging. All the phases of cell cycle and migration can be traced live in developing fish • **High-resolution and real-time imaging techniques** and methodologies established for live whole organism detection of biologically relevant dynamics in embryos, larvae and adults • **Availability of numerous transgenic fish, reporter lines and brain atlas** to identify various brain domains, precursor cells and differentiated cell types or to interrogate and manipulate signaling and cell behaviours • **Highly characterized behavioural readouts** and innovative methodologies are being established to quantitative assess epileptogenesis and cognitive skills in young larvae and profile zebrafish MCD disease models in a time window of less than 5 days, employing a relatively large sample size • **Broad conservation of developmental biology dynamics and signaling pathways** during embryogenesis also in the telencephalon • **High tissue regeneration** potential (including within the forebrain) • **High genetic homology** with humans (> 70% of human protein-coding disease genes have an orthologue in zebrafish), which is beneficial for mutant generation and disease modeling • **Genetic redundancy** resulting in some cases in the presence of more than one ortholog for one single human gene, often useful to overcome embryo lethality observed in mouse mutant of the single gene • **Availability of many mutant lines**, whose phenotype characterization at the early stage of development is faster compared to mammals • **Well-established genetic manipulation** to generate both transient and stable lines through a variety of genome editing approaches (*e.g.* CRISPR/Cas9, ZFNs, TALENs) • **Possibility of large screening** of mutant fish in F0 animals (without the expensive and time-consuming establishment of F1-F2 lines) • **Late development of the BBB** (blood-brain-barrier) allows easy and possibly **not-invasive uptake of active compounds**, reducing costs and animal burden normally associated with large drug screening in mammals. • **Alternative and informative model** for the modeling of human diseases that help optimizing reducing the use of larger animals and invasive procedures associated with animal distress, in line with the EU Directive 2010/63/EU and the 3R principle of animal experimentation	• **Genetic redundancy** can negatively impact on gene silencing and results’ interpretation in the context of human diseases modeling • **Variability in telencephalon development** compared to mammals and lack of a general consensus on the homologous region of forebrain and telencephalon regionalization between zebrafish and other vertebrates • **Absence of a laminar cortex** and cell type enrichment typical of mammals and gyrencephalic species • **Limitation in modeling neurodegeneration** due to remarkable ability to regenerate injured brains • **High costs for maintenance and experimentation compared to simpler invertebrate models** (*e.g. C.elegans*) • **Low degree of imbreeding** can result in high inter-individual variability and challenge data interpretation. First inbreed lines have been however generated and can be favoured in genetic studies

***In vitro models (iPSCs and organoids)***	

• **Renewable source of healthy cells and tissues** with varied differentiation potential into any of the three germ layers (*i.e* endoderm, mesoderm and ectoderm) for therapeutic purposes • **Multiple organ-specific cell types** can be easily accessible which are spatially organized in 3D-shape in organoids • **Minimal invasiveness** of the procedures required to collect donor somatic cells (*e.g.* skin fibroblasts, blood samples) • **Biobanking and safe and efficient autologous transplantation** for regenerative and personalized medicine, reducing risk of rejection by the immune system • COs can be used to study **network activity** • **Recapitulation of human physiology** (regionalization, cell types, cytoarchitecture in COs) and patient-specific traits • **Useful ethical alternative** to human embryonic stem cells (ESCs) to study human developmental processes *in vitro* • **Efficient** (about 40%), **rapid** (few weeks) and **safe** due to recent consolidation of genome editing technologies (*e.g.* RNA-mediated reprogramming, Cas9/CRISPR, piggyBac transposase) • **Lack of ethical implications** make these models useful alternative to reduce animal experimentation in respect to 3R principle (reducement, replacement and refinement)	• **Low reprogramming efficiency** of iPSCs (0.1 to 3%) due to residual epigenetic memory of the tissue from which they were derived • **High cost** for reprogramming and maintenance • **Human genetic heterogeneity** requiring rigorous genetic background characterization for disease modelling uses • **COs growth and maintenance** should be improved by overcoming the lack of sufficient nutrients and oxygenation • **Difficulty in recapitulating mature human brains** with COs that can be addressed by developing vascularized organoids that more faithfully recapitulate physiology • **Low efficiency of regional cerebral organoid differentiation** (e.g. dorsal or ventral forebrain) • **Tedious sample preparation** for imaging studies • **Limited protocol-development of co-cultures** with other cell types to model complex neurological disorders and recapitulate disease progression (*e.g.*excitatory neurons co-cultured with inhibitory neurons) • **Limited disease-modeling recapitulation for complex interactions of multiple genetic and environmental factors**

Besides the ease in management and the speed of breeding and development, many other unequaled factors contribute to springboard zebrafish for neurobiology. Transparent embryos (White et al., 2008; Antinucci and Hindges, 2016) eliminate the need for invasive procedures to manipulate and observe live events during embryogenesis, impossible in mammalian systems. Together with the embryo clarity, the continuous improvements of imaging tools of high-precision now facilitate further the investigation of brain development and function and allow to image even whole adult nervous system without invasive methodologies (Ahrens et al., 2013; Deán-Ben et al., 2016; Cong et al., 2017; Symvoulidis et al., 2017; Hontani et al., 2022). These features, in combination with an arsenal of transgenic lines marking cells from various growing brain territories of the forebrain (which reaches less than 300 μm in young larvae), i.e., main neuronal progenitors such as RG as well as differentiated telencephalic neurons (Bernardos and Raymond, 2006; Mione et al., 2008; Lal et al., 2018) permit to trace proliferative, neurogenic and migratory behaviors; potentially in the whole-brain (Mione et al., 2008; Novorol et al., 2013). Tools to image and quantify sub-cellular dynamics relevant to nervous system development and function are also used (Vacaru et al., 2014; Bercier et al., 2019; Asakawa et al., 2020; Lauri et al., 2021). Furthermore, study design, as well as results interpretation in zebrafish, are further supported by up to date databases, and methods for mapping molecular, cellular, and morpho-anatomical aspects of brain development available for zebrafish (Randlett et al., 2015; Gupta et al., 2018; Kunst et al., 2019).

In addition, the presence of genes in zebrafish which are orthologous to their human counterparts (Howe et al., 2013) is key for the generation of genetic models of diseases. Of note, the presence of more than one gene in zebrafish which is orthologous to a single human gene (genetic redundancy) - and which likely evolved different functions in the fish- is often beneficial to overcome the lethality observed by knocking out the single orthologous gene in mice (Kurahashi et al., 2005; Pei and Strauss, 2013). Furthermore, mutants deriving from large genetic screenings are available in zebrafish and for other laboratory teleosts, which help elucidate signaling, molecules and mechanisms involved forebrain patterning and regionalization (Schier et al., 1996; Loosli et al., 2000; Kitagawa et al., 2004). Adding to this, nowadays the generation of disease models in zebrafish is facilitated and accelerated by cutting-edge CRISPR-Cas methods (Hwang et al., 2013; Ablain et al., 2015; Varshney et al., 2015). This method can reach such high efficiency in this small freshwater fish to even allow to bypass the generation of stable genetically modified lines. These can be expensive, laborious, time-consuming and even prohibitive for some research groups. Therefore, while having the limitations of mosaic and transient models, the analysis of gene function in embryos, larvae, and adults of the F0 generation (named “crispants”), which can resemble null mutants, is permitting valid functional analyses in large settings (Hoshijima et al., 2019; Kroll et al., 2021; Masselink, 2021). The speed by which data are generated in this way is not currently achievable in mice models.

Moreover, advanced genome editing techniques with nucleotide precision recently adapted for zebrafish (Rosello et al., 2021) could even allow the generation of patient-specific mutants in the near future. It should also be added that a set of well-established behavioral assays is well characterized to monitor and classify cognitive tasks and epileptogenic responses in young zebrafish larvae (Orger et al., 2004; Levin and Cerutti, 2009), together with the use of *in vivo* reporters for brain activity and of electrophysiology protocols to record spontaneous epileptic events in mutants (Rosch et al., 2018; Yaksi et al., 2021). The establishment of reporter lines for many intracellular events, as diverse as calcium fluxes (Akerboom et al., 2012), pERK activity (Wong et al., 2019) or GTPases-mediated dynamics (Lauri et al., 2021) as well as of optogenetics (Simmich et al., 2012; Asakawa et al., 2020) are ideal for interrogating brain development and activity in healthy and disease fish models (Turrini et al., 2017; Asakawa et al., 2020).

Lastly, because a blood-brain barrier is fully formed only in relatively old zebrafish larvae, the uptake of CNS-directed drugs in the embryos and young larvae is possible via intraperitoneal injection or directly by dissolving the desired compound in the raring water (Quiñonez-Silvero et al., 2020). This possibility, together with the high availability of embryos per single fertilization event, reduces experimental times and cost for large screening and more invasive procedures associated with mammalian models; making zebrafish ideal for this purpose even in company-based settings (Ali et al., 2012; Chakraborti et al., 2016). In this context, it is worth considering that according to the EU Directive 2010/63/EU on animal experiments, early free-feeding embryos and larvae are classified as equivalent to *in vitro* systems. Thereby, even the costs, time and administrative load associated with the lengthy process to obtain authorization for animal experiments is easily reduced when working with zebrafish.

When applied to newly discovered and unknown MCD, these distinctive features can serve to get the first insights into the pathogenic mechanisms of disease and strategize large animal experiments. Of note, this scheme is in full agreement with the 3R principle (Sneddon et al., 2017), which advocates the use of alternative experimental models to reduce the overall animal number and burden associated with *in vivo* experiments, i.e., disease modeling.

### Limitations

Clear limitations need to be recognized when using zebrafish to model human brain malformations (Table 3). As discussed, we currently have limited knowledge on the complexity, developmental and functional potential of teleosts and zebrafish forebrain, which does not account for all the constituent progenitor cell types of the human neocortex and does not develop a laminated cerebral cortex. From here, an obvious limitation exists when dissecting disease mechanisms underlying cortical folding defects, which could underly *ad hoc* processes only present in gyrencephalic vertebrates. Clearly, to better design and interpret zebrafish MCD models that are generated, more thorough comparative investigations are needed. Furthermore, the occurrence of neurogenic activity in the adult fish brain and regeneration upon injury can help us to gain insights of translational value into possible triggers of neuronal regeneration in vertebrates (Strobl-Mazzulla et al., 2010; März et al., 2011; Schmidt et al., 2013; Barbosa et al., 2015). However, the specific regenerative mechanisms in place might alter the phenotype obtained in fish models of brain disease. Moreover, the presence of more than one orthologous gene for some human candidate genes potentially involved in disease might make it necessary to establish a double gene manipulation (e.g., double knockdown approach) to achieve a valid model recapitulating the human disease. While -as discussed- the large number of embryos and larvae available per experiment is ideal for statistical evaluation of the results, little inbreeding in zebrafish lines can underly a high variability in the response of individual animals to genetic manipulations and behavioral assays (Fitzgerald et al., 2019). On the other hand, the use of medaka is ideal for genetic studies, as inbreed, fertile and vital lines have been established in this fish (Murata et al., 2019) and, more recently, also in zebrafish (LaFave et al., 2014).

### Examples of Disease Modeling and Translational Value

Even with these limitations in mind, it is clear that the combination of a set of specific features lacking in larger animal models makes zebrafish an extremely resourceful model system. This explains the popularity of zebrafish in translational research to (1) establish fast quantitative platforms to screen and investigate the increasing number of candidate pathogenic variants, (2) perform an initial investigation of the underlying mechanisms of disease, and (3) examine potential therapeutic targets. Indeed, from a translational point of view, zebrafish models are useful to validate previously undiscovered rare neurodevelopmental diseases within integrated functional genomics pipelines. Here, different pathogenic variants are first identified via NGS screening in patients, their differential impact on cellular and molecular events is often assessed in *in vitro* systems. Zebrafish is widely used to map the effect of these variants on embryogenesis and neural development and to connect back to the clinical features observed in humans.

To this date, numerous examples exist of functional validations of candidate pathogenic variants in neurodevelopmental diseases involving multi-modal functional genomics approaches (Sakai et al., 2018; Lauri et al., 2021). For instance, recent patients’ genomic sequencing, *in vitro* and *in vivo* zebrafish modeling was carried out in a single study to assess the pathogenicity of newly discovered mutations in the aminoacyl-tRNA synthetase-encoding *VAR2* gene. Mutations in this gene lead to microcephaly, intellectual disability, and epilepsy (Siekierska et al., 2019). A *var2* knock-out zebrafish model was generated within this integrated framework, and demonstrated the causal link of loss of function mutations with major features of the neurodevelopmental disorder observed in patients, even at the level of neurobehavioral traits. Indeed, specific assays could be used to score the phenotypes in zebrafish larvae. In addition, the assessment of the complementing potential of various mutations in this zebrafish model was helpful in differentiating the pathogenic contributions of the variants and to highlight the underlying genetic mechanism in the vertebrate embryo (Siekierska et al., 2019). Many zebrafish models have proven their utility in validating genes and variants affecting proteins participating in various intracellular processes and to model aspects of MCD diseases, directly complementing mammalian models. An extensive account is reported in Table 2. As an example, a recent genome-wide and exome screening identified a truncated mutation with loss of function of MAP11 (microtubule associated protein 11) as cause of recessive primary microcephaly. While the role in mitotic spindle dynamics and proliferation was proven *in vitro*, the pathogenicity of the loss of function generating microcephaly was demonstrated using zebrafish mutants, that complemented and validated the finding (Perez et al., 2019). Thanks to these integrated approaches, overlooked genes and mechanisms causing MCD are emerging. For instance, recently, we identified a previously unknown Golgipathy involving dominant mutations in the new disease gene encoding for the small GTPase ARF3. We demonstrated the validity of using zebrafish to recapitulate the microcephaly observed in patients and to validate the disruptive effect on the Golgi stability *in vitro* and in embryos (Fasano et al., 2021). Dedicated studies assessing and differentiating the mechanisms of previously discovered genetic lesions using zebrafish models also exist. For example, mutant and morpholino-induced knock-down of a number of genes involved in primary microcephaly have been described, which recapitulated the basic centrosome defects. The data also proved an increased rate of proliferative and dividing cells failing to progress through prometaphase in the neuroepithelium of developing zebrafish (Novorol et al., 2013; Table 2). It is worth mentioning that, thanks to fish *in vivo* models, intracellular trafficking and ECM deposition defects were demonstrated to be the cause of a heterogeneous group of dystroglycanopathies leading to dystrophies associated with retinal defects and lissencephaly (Lin et al., 2011; Stevens et al., 2013), while the embryo lethality observed in the corresponding mouse model of these diseases had previously hindered their experimental usefulness (Kurahashi et al., 2005). This exemplifies further the complementary use of zebrafish in the field.

Besides microcephaly, even more complex aberrations at the level of white matter formation and connectivity, as well as telencephalon cell positioning and global organization seen in patients with TSC, can be modeled and studied in zebrafish *tsc2* mutants. This can be done at the cellular and neurobehavioral level (i.e., assessing epileptogenic output) (Kim et al., 2011b; Scheldeman et al., 2017; Kedra et al., 2020), and indicates deep conservation of the fundamental processes of telencephalon ontogeny and function in vertebrates, and the usefulness of zebrafish in modeling MCD.

Generally, examples exist also of direct use of zebrafish embryo models of rare (neuro)developmental diseases in the search of possible candidate drugs to counteract impaired signaling cascades [such as Ras/MAPK (Bobone et al., 2021)] or ameliorating seizures. (Baraban et al., 2013; Dinday and Baraban, 2015) performed an extensive screening to identify antiepileptic molecules effective on zebrafish model of Dravet Syndrome. (Griffin et al., 2017) took further advantage of the model, showing case of a *in vivo*, rapid path toward the *off-label* employment of lorcaserin. This FDA-approved compound antagonizing serotonin signaling pathway was transferred from preclinical discovery to potential clinical treatments of Dravet syndrome. From here, the compassionate use of commercial Belviq^®^ is now approved for a small population of children affected by the syndrome, who successfully exhibit a reduction of generalized tonic-clonic events.

## Induced Pluripotent Stem Cells and Organoids-Based Models of Human Corticogenesis

To fully exploit the unique features of zebrafish (and more generally teleosts) for functional validation and classification of rapidly emerging heterogeneous MCD in humans, experimental designs, questions, and data interpretation in these models should be carefully confined by the limitations discussed. In translational workflows dedicated to solving the underlying biology of MCD *in vivo* zebrafish models should be complementary to other feasible alternatives closer to large mammals and humans (Figure 1). Of particular interest in this sense and complementing the popularity of gyrencephalic mammals, is the establishment of *in vitro* human models of neural proliferation and differentiation using induced pluripotent stem cells (iPSCs), as well as iPSCs-derived COs for better recapitulating the structural and functional complexity of the human brain (Figure 1 and Table 1). These advances have been possible thanks to the studies of the early 20th century on neural induction from tissues of animal models and capitalize on the cell-autonomous competence of progenitor stem cells, especially in organizing anterior neural tissue (Lancaster et al., 2013; Paşca et al., 2015). iPSCs can be obtained from somatic cells by induction of known factors that reprogram the adult tissue toward different cell/tissue fate, including cortical neurons. COs have been more recently established (Lancaster et al., 2013) as 3D-self-oganizing cerebral tissue cultures derived from stem cells (i.e., iPSCs) and can be useful to study all the different processes of cortex formation, including interneurons migration (Lancaster et al., 2013).

### Advantages

iPSCs can be an informative and renewable source of cells to model human and mammalian neurogenesis. Their use already helps reduce the number of animals and the possible distress caused by studying cortical development and function in mammals. Furthermore, a precious source of information derives from iPSCs directly obtained from residual bioptic material of rare patients. In addition, among the main advantages of using iPSCs to obtain simplified models of neural networks (Table 3), one should note: (1) the easy accessibility to all cell types obtained, (2) the relatively fast timing of circa 50–60 days to obtain mature neurons, and (3) the possibility to analyze cell morphology and dynamics with relatively user-friendly tools (i.e., imaging tools and softwares). The procedure required to obtain cells to generate iPSCs is only minimally invasive in humans and animals and the use of iPSCs replaces the need to employ directly human embryonic stem cells, for which bioethical concerns exist. Another advantage is the optimization of gene modifications, which include the CRISPR/Cas approaches and facilitates the study of gene function (Ben Jehuda et al., 2018). Moreover, patients-derived clones, which are also genetically modified for therapeutic purposed, can be stored via biobanking and available at modern healthcare institutes for safe autologous transplantation, and regenerative and personalized approaches (Huang et al., 2019; Palechor-Ceron et al., 2019).

Nevertheless, these cells are in a 2D environment that hardly recapitulates the elaborated, multi-layered architecture of the human brain. They might show a low programming efficiency and can be very expensive to obtain. 3D systems, such as embryoid bodies (EB) first and cerebral organoid models (COs) later, are obtained via different protocols (Chiaradia and Lancaster, 2020) and they mostly rely on the ability of self-organization of stem cells in rosettes and on the stochastic cell differentiation within a homogeneous group of cells (Weiss and Taylor, 1960). Potentially, COs generated from normal or patient-derived iPSCs can better recapitulate the human cell type compendium in the cortex (Eze et al., 2021) (Table 3) and the impact of genetic mutations on human cortical neurogenesis as compared to mice models (Bershteyn et al., 2017; Iefremova et al., 2017; Li et al., 2017a; Kyrousi et al., 2021). Compared to iPSCs, COs can mimic the different specifications of the CNS (such as forebrain organoids). Developmental tissue organization along the axes (including the presence of VZ and of multiple layers) and the birth of various cellular identities at different stages of neurogenesis and differentiation such as RG, IPs, excitatory and inhibitory neurons and glia in defined temporal sequences in COs broadly match those observed in the human fetus (Shi et al., 2012). Furthermore, neuronal ensembles developing COs that self-organize establish connectivity in a three-dimensional setting and subdomains identity (Lancaster et al., 2013). Over a period of more than 9 months, relatively mature features, such as the presence of dendritic spines and active neuronal networks, have been documented in COs by a high-throughput single-cell transcriptional profiling method and extracellular recordings with high-density silicon microelectrodes (Quadrato et al., 2017). Moreover, neurons within the established COs are electrically active (Lancaster et al., 2013; Lancaster and Knoblich, 2014). The spontaneous Ca2 + spikes observed under baseline conditions increase upon stimulation with glutamate and decrease by inhibiting action potential, which demonstrates their dependence upon neural activity (Lancaster et al., 2013; Lancaster and Knoblich, 2014).

Comparative iPSCs and COs preparations are also useful to understand the ontogeny of species-specific similarities and differences in proliferative potential, cortex size and complexity, underlying the variability in cognitive specialization across taxa (Espuny-Camacho et al., 2013; Mora-Bermúdez et al., 2016). For instance, the timing of cortical progenitor proliferation and cell-type specification is most likely based on genetic control in different species, as evinced by experiments with mixed progenitor cell culture assays between and within species (human and macaque) (Otani et al., 2016). From this study, species-specific programs regulating the modality of progenitor expansion also emerge, which ultimately determines differences in the cortex size between various primates and mice (Otani et al., 2016). Comparative COs studies also show that protracted neurogenesis might be a driving factor of human neocortex expansion (Espuny-Camacho et al., 2013; Stepien et al., 2021). This kind of information could be the ground to interpret various available models of human MCD (Li et al., 2017b).

### Limitations

Given the increase in the use of COs in biomedical science, one should also consider that, notwithstanding these key features, at present, the extent to which brain organoids recapitulate the cellular diversity, regional complexity, and circuit functionality of the brain remains poorly addressed. COs lack macroscale patterning, microglia, and healthy vascularization. In addition, they are limited in the degree of neuronal connectivity and relationship with other tissues (Table 3). Efforts are being made to better characterize the features of COs compared to the mammalian cortex, and new methods are being developed to counteract these significant limitations. Notably, the first COs have been obtained by self-patterning, but most recently, region-specific brain organoids were derived using extrinsic signals known to pattern brain regions during embryogenesis (Susaimanickam et al., 2022). By modulating the concentrations of the morphogens, researchers obtained COs of the hippocampus (Paşca et al., 2015; Sakaguchi et al., 2015) or midbrain (Qian et al., 2016). Further, by modulating Sonic Hedgehog levels it was possible to pattern forebrain organoids into dorsal and ventral mid-domains (Cederquist et al., 2019) and addition of BDNF, GDNF and ascorbic acid can lead to differentiation toward the midbrain and brainstem identity (Eura et al., 2020). Of note, (Pellegrini et al., 2020) recently established choroid plexus-forming organoids producing cerebrospinal fluid, a source of developmental factors to sustain COs maintenance. The availability of this CNS barrier *in vitro* also holds promises to study drug delivery into the human brain (Pellegrini et al., 2020). To achieve a higher and more physiological complexity, in recent studies, human microglia has been xenotransplanted into COs, leading to an acceleration of the synchronized oscillatory network activity (Popova et al., 2021), while vascularized brain organoids start to be engineered to more faithfully recapitulate human condition (Cakir et al., 2019). On the other hand, *in vivo* organoid transplantation can provide a better organismal context, such as supplying inductive microglia and interaction with other cell types.

In addition, it is worth considering the potential threat of the high variability existing between different preparation of COs, which raises concern about the validity of the conclusion drawn with respect to human brain development and disease (Quadrato et al., 2016, 2017; Velasco et al., 2019). Nevertheless, single-cell RNA sequencing from various cells of different COs preparations show that they can have relatively mature features, including spontaneously active neuronal networks (Quadrato et al., 2017). COs can potentially reproduce the complexity of the central nervous system developed in the embryo in terms of the richness of the cell types produced with a variability that would also be observed *in vivo* (Velasco et al., 2019). Single-cell RNA analysis generating an atlas of early human brain development recently demonstrated that human specific cortical progenitors not found in mice models are present in COs, however they display low fidelity to neuroepithelial and early radial glia cell types, but improves as neurogenesis progresses (Eze et al., 2021). Moreover, (Ziffra et al., 2021) demonstrated a limited contribution of epigenetics to the patterns of cell type diversity and cell fate specification of COs as compared to human cortex. Lastly, the poor control over the self-organization and differentiation mechanisms resulting in heterogeneous preparations and inter-lab variability is being addressed by a chemical and physical engineering methods that better control and advance *in vitro* cortical programming and morphogenesis. For instance, a sequence of exposure to Wnt inhibitors first and to Wnt molecules later seems to efficiently guide dorsal forebrain commitment (Chiaradia and Lancaster, 2020). Furthermore, microfluidic devices are being engineered to reproduce morphogen gradients quantitatively (Garreta et al., 2021). Moreover, multi-domains cerebral tissues grown on a chip have been established that show functional interconnectivity and can model epileptic discharges more accurately (Saberi et al., 2022). More biotechnological efforts in this direction are indeed necessary to provide features able to unveil the fidelity and robustness of COs as a model for cortical development.

### Examples of Disease Modeling and Translational Value

A considerable amount of neurodevelopmental disorders has been modeled by region-specific brain organoids, i.e., microcephaly, lissencephaly, Rett syndrome, schizophrenia, autism spectrum disorder, Pelizaeus-Merzbacher disease, Timothy and Prader-willi syndrome. These models demonstrated their usefulness in unveiling patho-mechanisms and potential therapeutic opportunities (Lu et al., 2022; Susaimanickam et al., 2022). Another relevant example of how COs can capture clinically relevant features originating from altered cerebrum development and function is the study on Autism Spectrum Disorder (ASD) (Paulsen et al., 2022). Here, COs were used to identify developmental abnormalities resulting from haploinsufficiency in three ASD risk genes from different donors and to evaluate phenotypic convergence (Paulsen et al., 2022). Other studies where brain organoids were useful to understand the molecular basis of human interneuron migration have been performed by Birey and colleagues (Birey et al., 2022) by using assembloids obtained from the integration of cortical and ventral forebrain organoids and unveiling details of cortical interneuron migration defects in Timothy syndrome. Besides the zebrafish approaches discussed (Table 2), iPSCs and COs developed from patients with MCD are already contributing to the understanding of specific diseased cortical networks (Table 2). For example, neuronal 3D cultures from a patient exhibiting primary microcephaly due to *ASPM* mutation more faithfully recapitulated the cerebral size reduction seen in the patient as compared to animal models (Pulvers et al., 2010). This model also showed characteristic neurogenic, structural, and network activity defects (Li et al., 2017a). Similarly, COs from patients with *CDK5RAP2* mutations affecting centrosomes dynamics recapitulated the microcephaly which was difficult to analyze in mice models and provided valuable insights into the disease mechanism underlying altered neurogenesis (Lancaster et al., 2013). Furthermore, IPSCs-derived neurons and forebrain organoids with *LIS1* mutations exhibited proliferative defects, resembling also the zebrafish model (Iefremova et al., 2017); while the molecular mechanisms underlying focal dysplasia and tuberous sclerosis due to mutations in *TSC1* and *TSC2* have been studied in 2D and 3D cortical systems from human brain (Marinowic et al., 2017; Blair et al., 2018). Complementing the models available in zebrafish (Kedra et al., 2020) (Table 2), these studies showed an induced mTOR signaling and sustained gliogenesis as major contributors to the disease (Marinowic et al., 2017; Blair et al., 2018). Moreover, COs models of how migratory defects impact the correct formation of the cortex are also available (Buchsbaum et al., 2020).

From a more translational point of view, the use of brain organoids as model systems for drug development has been largely proposed (Lancaster et al., 2013; Li et al., 2017b; Klaus et al., 2019; Costamagna et al., 2021; Matsui and Shinozawa, 2021; Xu et al., 2021; Lu et al., 2022; Susaimanickam et al., 2022). Nageshappa et al. (Nageshappa et al., 2016) demonstrated the use of cortical neurons derived from human iPSCs-based model of MECP2 duplication for the identification of the histone deacetylase inhibitor, the epigenetic modifier NCH-51, as a potential clinical candidate to rescue the altered neuronal phenotype characterized by increased activity frequency, synchronized bursts, and more active synapses found in MECP2 patients. CDLK5-deficient human organoids showing increased frequency and synchrony of spikes (neuron hyperexcitability), a hallmark of the intractable early-onset epilepsy affecting pediatric patients were employed for drug screening application. The calcium-imaging screening in the model identified promising compounds of different classes (inhibitor of muscarine receptors, Notch inhibitor, GSK3 inhibitor and hyperpolarization-activated cyclic nucleotide-gated channel blocker) as potential therapeutic target for pediatric patients with defective CDLK5 (Shcheglovitov and Peterson, 2021).

## Conclusion

The ontogeny, morphogenesis, and structural/functional identity of the forebrain domains, and especially of the pallium, from which the cortex and neocortex originated, arose via divergent paths in vertebrates which likely represent key events for multiple adaption strategies throughout evolution. Clearly, the neocortex and its expansion are only visible in a subset of mammals. Its radial construction is achieved via a delicate series of events and precursor cell types, whose proliferative potential and neurogenic timing most likely shapes the differences observed in size and complexity across mammals. The alteration of the fine-tuning of these processes leads to rare conditions with heterogeneous causes and severe clinical consequences. Rapid protocols for effective modeling of these conditions, which are increasingly being reported, are pivotal to tackling the underlying mechanisms and prompt patient care. On the other hand, increasing experimental evidence from the field of comparative neurobiology shows that the teleost brain, considered “simple” for long time, exhibits high cognitive functions, some of the underlying core cell types, development strategies and circuits which are closely related to those build within mammalian pallium derivatives. However, analysis of the extent of similarity and shared ancestry between the pallium ontogeny, morphology, and function in teleosts and amniotes (and gyrencephalic) mammals just began. From a technical point of view, compared to classical and innovative mammalian models, in both the context of brain EvoDevo and disease, teleosts offer a distinctive readiness for generating affordable models with a broad and fast interrogation capacity of nano-scale dynamics within precursor cells and up to neuronal circuits logics of entire brain territories at the whole-organism scale. Furthermore, quantifiable behavioral readouts can be performed simultaneously, creating an ideal setting for scalable preclinical drug testing. Specifically, for disease modeling, limitations exist, however, when researching biological processes which could be altered in MCD using zebrafish and other teleosts, given the differences discussed so far and the clear lack of a *bona fide* cortex in this species. Not with understanding these differences, fish have indeed already demonstrated their usefulness in speeding up functional validations of new MCD-causing variants and disease genes. In this context, they can often model basal defects in brain/forebrain development, at the level of proliferation, migration and connectivity, which recapitulate those found in patients (Table 2). It is without a doubt that zebrafish work has already benefited our understanding of rare neurodevelopmental diseases. However, the speed by which teleosts are being used, calls for a careful interpretation of the data obtained in these models, which should be assessed against complementary systems more closely resembling human cortical features. Mammalian 2D and 3D neuronal cultures -especially if patient-derived- begin to provide interesting possibilities. We are at the beginning of a new era for brain disorder modeling. Understanding the extent to which small vertebrate fish and 3D organoid cultures can be used as high-fidelity/informative systems recapitulating human brain development is compulsory. As shown, examples of successful *in vivo* fish models for MCD exist, which expand the possibilities offered by COs and validate the use of teleost fish in the translational research of MCD. Complementary studies combining “fish and dish”-based brain models are accumulating, which demonstrate how they may be extremely compelling to dissect different aspects of human rare disorders (Ayala-Nunez et al., 2019; Pini et al., 2020) and to counterbalance possible pitfalls of the single models (Hengel et al., 2020). A robust integration of patients’ genomic sequencing and functional investigations in zebrafish, iPSCs and organoids exhibiting diverse and unbeatable advantages promises to be a simplified yet efficient multimodal paradigm of contemporary translational research on MCD.

## Author Contributions

AL conceived the manuscript and generated the illustration. AL, GF, and CC wrote the manuscript. MT and BD revised the final manuscript. GF and AL conceived and generated the tables. All authors contributed to the article and approved the submitted version.
